# Supplementary material for: Microbial Feast or Famine: dietary carbohydrate composition and gut microbiota metabolic function
Source: bioRxiv. 2025 Oct 28:2025.10.27.684932. Preprint. [Version 1] doi: 10.1101/2025.10.27.684932 (PMC12636310; doi:10.1101/2025.10.27.684932)
Supplement: Supplement 1 [file media-1.docx]

**Supplementary Material**

**
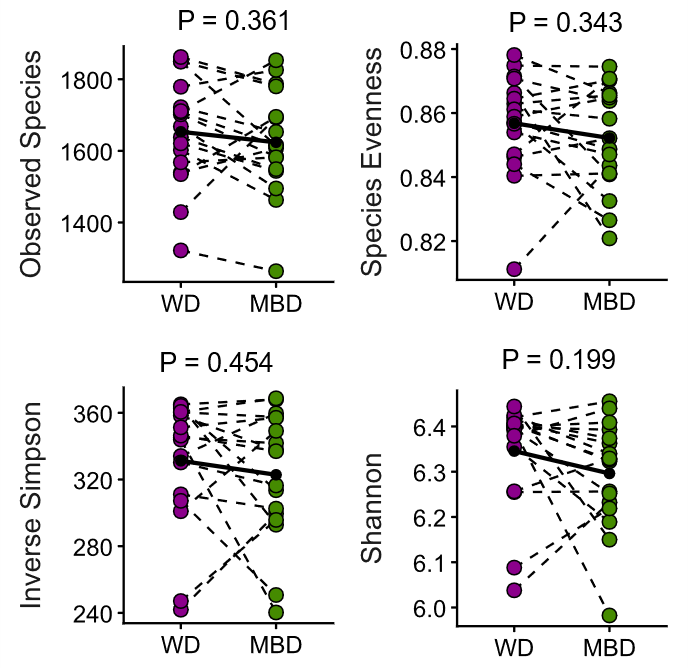
**

**Supplementary Figure 1. Alpha-diversity metrics.** Measured alpha-diversity metrics observed species, evenness, Inverse Simpson, and Shannon diversity were not significantly different between the WD and the MBD. N = 17.
